# p518, a small *floR* plasmid from a South American isolate of *Actinobacillus pleuropneumoniae*

**DOI:** 10.1016/j.vetmic.2017.04.019

**Published:** 2017-05

**Authors:** Giarlã Cunha da Silva, Ciro César Rossi, Mateus Ferreira Santana, Paul R. Langford, Janine T. Bossé, Denise Mara Soares Bazzolli

**Affiliations:** a Laboratório de Genética Molecular de Bactérias, Departamento de Microbiologia, Instituto de Biotecnologia Aplicada à Agropecuária (BIOAGRO), Universidade Federal de Viçosa, Viçosa, Brazil; b Section of Paediatrics, Department of Medicine, Imperial College London, St. Mary’s Campus, London, UK

**Keywords:** Antibiotic resistance, Florfenicol, Respiratory tract, *Pasteurellaceae*

## Abstract

A small (3.9 kb) plasmid (p518), conferring resistance to florfenicol (MIC >8 μg/mL) and chloramphenicol (MIC >8 μg/mL) was isolated from an *Actinobacillus pleuropneumoniae* clinical isolate from Southeastern Brazil. To date, this is the smallest florfenicol resistance plasmid isolated from a member of the *Pasteurellaceae.* The complete nucleotide of this plasmid revealed a unique gene arrangement compared to previously reported florfenicol resistance plasmids found in other members of the *Pasteurellaceae*. In addition to the *floR* gene and a *lysR* gene, common to various florfenicol resistance plasmids, p518 also encodes *strA* and a partial *strB* sequence. An origin of replication (*oriV*) similar to that in the broad host range plasmid, pLS88, was identified in p518, and transformation into *Escherichia coli* MFD*pir* confirmed the ability to replicate in other species. Mobilisation genes appear to have been lost, with only a partial *mobC* sequence remaining, and attempts to transfer p518 from a conjugal donor strain (*E. coli* MFD*pir*) were not successful, suggesting this plasmid is not mobilisable. Similarly, attempts to transfer p518 into a competent *A. pleuropneumoniae* strain, MIDG2331, by natural transformation were also not successful. These results suggest that p518 may be only transferred by vertical descent.

## Highlights

• Smallest *floR* plasmid in the *Pasteurellaceae*.
• Unique arrangement with complete *strA*, but partial *strB* and *sul2* sequences.
• Loss of mobilisation genes indicated by partial *mobC*; no other *mob* genes.
• Not transferrable by conjugation or natural transformation.

## 1 Introduction

Porcine pleuropneumonia is a severe respiratory disease causing extensive economic losses in the swine industry worldwide, and *Actinobacillus pleuropneumoniae*, a member of the *Pasteurellaceae* family, is the main etiologic agent of this disease (Gottschalk, 2012). Reductions in morbidity and mortality can be achieved to some degree using good husbandry practices and vaccination, however subclinical and acute disease may still occur (Gottschalk, 2015). Treatment with antimicrobial agents is necessary to limit the spread and severity of disease in the case of clinically active infection.

In many countries, the most commonly used antimicrobial agents for treating porcine respiratory disease are tetracyclines, macrolides/lincosamides, aminoglycosides, beta-lactams, and trimethoprim/sulphonamides (Guardabassi et al., 2008). There is currently no information regarding relative amounts of different antimicrobial agents used for veterinary treatments in Brazil (Bokma et al., 2014), however florfenicol, an antimicrobial agent used exclusively in veterinary medicine, has only been used in Brazil since 1996 (SINDAN, 2013). Levels of resistance to florfenicol are still very low for *A. pleuropneumoniae* in most countries (Dayao et al., 2014, Garch et al., 2016, Vanni et al., 2012), except in Korea where extensive use of this antimicrobial agent has led to resistance in 34% of isolates tested (Yoo et al., 2014). *A. pleuropneumoniae* isolates resistant to florfenicol have been shown to harbour the *floR* gene encoding a chloramphenicol/florfenicol efflux pump, FloR (Kucerova et al., 2011, Yoo et al., 2014, Bossé et al., 2015). The prevalence of florfenicol resistance amongst *A. pleuropneumoniae* isolates in Brazil has yet to be determined, however we recently identified a clinical isolate, MV518, from Southeastern Brazil that carries the *floR* gene, as identified by Resfinder analysis (Zankari et al., 2012) of the draft genome sequence (Pereira et al., 2015). Here we describe the isolation and complete nucleotide sequence of a 3.9 kb plasmid, p518, from *A. pleuropneumoiae* MV518.

## 2 Materials and methods

### 2.1 Bacterial strain and antimicrobial susceptibility testing

*A. pleuropneumoniae* MV518, a serovar 8 clinical isolate, was kindly provided by Microvet (Minas Gerais). Minimum inhibitory concentrations (MICs) for florfenicol and chloramphenicol were determined by the broth microdilution susceptibility assay, according to the CLSI VET01-A4 guidance (CLSI, 2013).

### 2.2 Sequence analysis and plasmid isolation

The whole genome sequence of MV518 (accession number JSVZ00000000), previously reported (Pereira et al., 2015), was analysed using ResFinder (Zankari et al., 2012) for identification of resistance genes, using a threshold of 98% identity and minimum length of 60%. Further sequence analysis was performed using BLASTn and BLASTx (http://blast.ncbi.nlm.nih.gov/Blast.cgi). Plasmid sequence alignments were performed using ClustalW (Larkin et al., 2007).

**Fig. 1:**
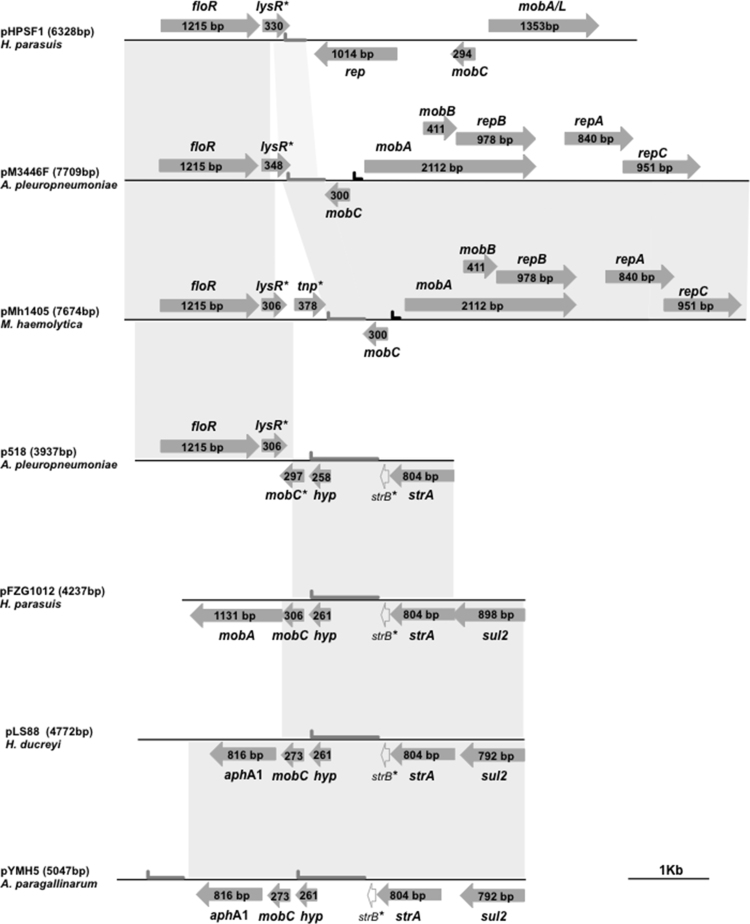
Schematic comparison of p518, a florfenicol resistance plasmid from *A. pleuropneumoniae* strain MV518, with other *Pasteurellaceae* plasmids. Open reading frames are indicated by arrows, with arrowheads showing direction of transcription (*floR*: florfenicol resistance; lysR*: partial *lysR* transcriptional regulator; tnp*: truncated transposase gene; *mob*, *mobA*, *mobB*, *mobC*, *mobA/L*: plasmid mobilisation; mobC*: partial *mobC* gene; *repA*, *repB*, *repC*: plasmid replication; *sul2*: sulphonamide resistance; *strA*: streptomycin resistance; strB*: partial *strB* streptomycin resistance; *hy*p: hypothetical gene). The predicted origins of replication (oriV; ) and transfer (oriT; ) are shown. Grey blocks between sequences indicate ≥ 98% nucleotide sequence identity. A distance scale in kb is shown.

Plasmid DNA was extracted from MV518 using the Qiaprep extraction kit (Qiagen). Inverse PCR was performed, using 1U of Platinum^®^ Taq DNA polymerase (Invitrogen) according to the manufacturer’s instructions, with outward facing primers (strA_I_F TAACGCCGAAGAGAACTGGG and strA_I_R AAGTTGCTGCCCCATTGACG) designed to amplify the plasmid sequence found between the 5′ and 3′ ends of the *strA* gene (see Fig. 1). The resulting amplicon was purified with the QIAquick PCR Purification Kit (Qiagen) and completely sequenced using a primer walking strategy. The 3937 bp sequence of p518 was manually annotated and deposited in Genbank under the accession number KT355773.

### 2.3 Transformation and conjugation

Initially, p518 was transformed into *E. coli* MFD*pir* (Ferrieres et al., 2010) by heat shock, with selection on Luria-Bertani agar plates containing florfenicol (10 μg/mL) and 0.3 mM diaminopimelic acid (DAP: required for growth of the MFDpir strain). The presence of p518 in transformants was confirmed by PCR amplification of a *floR* gene sequence using primers floR1 (5′-GCGATATTCATTACTTTGGC-3′) and floR2 (5′-TAGGATGAAGGTGAGGAATG-3′), and plasmid size was confirmed by linearisation with SacI. Subsequently, mobilisation of p518 from *E. coli* MFD*pir* into the plasmid-free, florfenicol sensitive *A. pleuropneumoniae* strain MIDG2331 (Bossé et al., 2016), was assessed in three separate experiments, using a previously described conjugation protocol (Bossé et al., 2009). For determination of plasmid uptake by natural transformation, three separate experiments compared the ability of either 1 μg of uncut purified plasmid p518, or 1 μg of control genomic DNA (carrying a *dfrA14* trimethoprim resistance gene inserted in the chromosome), to transform MIDG2331, as previously described (Bossé et al., 2014), with selection of transformants on BHI agar supplemented with 0.01% NAD and either 5 μg/mL florfenicol, or 10 μg/mL trimethoprim, as appropriate.

## 3 Results and discussion

Analysis of the draft genome of MV518 using Resfinder revealed a contig (Accession number JSVZ00000000, contig 40) containing the *floR* and *strA* resistance genes. When tested by the broth microdilution susceptibility assay (CLSI, 2013), strain MV518 had an MIC >8 μg/mL for both florfenicol and chloramphenicol, consistent with *floR* encoding a chloramphenicol/florfenicol efflux pump (Braibant et al., 2005).

A 3.9 kb plasmid, p518, was extracted from MV518 and the complete nucleotide sequence determined. Sequence analysis revealed that p518 appears to have arisen by recombination of a region encoding the 1215 bp *floR* gene and a 306 bp ORF showing similarity to predicted *lysR* genes, with sequences from a plasmid encoding an 804 bp *strA* gene followed by a truncated (98 bp) *strB* sequence (Fig. 1). Although the sequence containing the *floR* and *lysR* genes is common to numerous *floR* plasmids, including those found in members of the *Pasteurellaceae* such as pM3446F (accession number KP696484) from *A. pleuropneumoniae* (Bossé et al., 2015), and pHPSF1 (accession number KR262062) from *Haemophilus parasuis* (Li et al., 2015), the highest similarity (99% identity) over the first 1933 bp of p518 was seen with the *Mannheimia haemolytica* plasmid, pMh1405 (accession number AB621552) (Katsuda et al., 2012). The remaining sequence of p518 shows greatest identity (2003 of 2004 bp) to part of a 4237 bp plasmid from *H. parasuis*, strain FZG1012 (accession number HQ015158), which carries the resistance genes *sul2* and *strA*, as well as mobilisation genes *mobC* and *mobA* (Liu et al., 2011). This 2 kb sequence is also highly similar to regions of pYMH5, a 5047 bp plasmid from *Avibacterium paragallinarum* (Hsu et al., 2007), and pLS88 (accession number L23118), a 4772 bp plasmid from *Haemophilus ducreyi* (Dixon et al., 1994), having 2002 or 1994 identical bases, respectively (Fig. 1). Although only 14 bases of the *sul2* gene (3′ end), and 141 bases of the *mobC* gene (coding for the first 47 AAs), can be identified in the p518 sequence, the *strA* gene, as well as the broad-host-range origin of replication (*oriV*), are complete (Fig. 1). We confirmed the ability of p518 to replicate in another species by transforming it into *E. coli* MFDpir (Ferrieres et al., 2010).

Although a 297 bp ORF was identified which shares 100% identity over the first 142 bp with the *mobC* gene found in the other *Pasteurellaceae sul2*/*strA* plasmids described above, the remaining 155 bp do not match known *mobC* genes. It is likely that the recombination between the *floR*/*lysR* containing sequence and the *strA* containing plasmid resulted in an alternate stop codon for this ORF in p518, and it is not known if it encodes a functional protein. Similarly, the sequences of the *lysR* genes in the various plasmids shown in Fig. 1 all share a common 5′ end, but differ at their 3′ ends (suggesting different stop codons acquired following recombination), and it is not known if any of these genes encode a functional regulatory protein of the LysR family.

We tested the ability of p518 to be mobilised from the DAP-dependent *E. coli* donor strain, MFD*pir*, which encodes all of the genes required for generating conjugal transfer machinery (Ferrieres et al., 2010), into a plasmid-free recipient strain of *A. pleuropneumoniae*, MIDG2331 (Bossé et al., 2016), however we were unable to detect transconjugants containing p518. It appears that p518 is not capable of being transferred via conjugation, which is consistent with the lack of mobilisation genes in this plasmid.

As isolates of *A. pleuropneumoniae*, including MIDG2331, are known to be competent for natural transformation (Bossé et al., 2014), we investigated the possibility that p518 could be transferred horizontally by this mechanism. The sequence of this plasmid contains seven of the nine bases of the uptake sequence (ACAAGCGGT; bases in p518 are underlined) known to be required for efficient transformation of *A. pleuropneumoniae* (Redfield et al., 2006). A transformation frequency of ×10^−5^ was achieved using the control genomic DNA, whereas the p518 plasmid yielded no detectable transformants.

In summary, to the best of our knowledge, p518 is the smallest florfenicol resistance plasmid so far isolated from a member of the *Pasteurellaceae*, and this is the first report of florfenicol resistance in *A. pleuropneumoniae* in South America. Plasmid p518 shows a novel gene organization, sharing regions of high sequence identity with other *Pasteurellaceae* plasmids, suggesting that the *floR* gene may have been exchanged between different plasmids in closely related pathogens (and/or commensals) that share that same host environment. Despite the fact that we were not able to demonstrate horizontal transfer of p518 by either mobilisation or natural transformation, it is apparent from the conservation of the *floR*/*lysR* region in this and numerous other plasmids, that this is a highly recombinogenic sequence, and p518 could therefore act as a donor for spread of the *floR* gene into other, more mobile, plasmids. An antimicrobial susceptibility survey of *A. pleuropneumoniae* isolates in Brazil is required to determine the prevalence of resistance to florfenicol and other important antimicrobial agents, and continued surveillance to monitor spread of resistance is essential.

## Conflict of interest

We declare that we have no conflict of interest.

## Funding

This work was supported by Fundação de Amparo à Pesquisa do Estado de Minas Gerais – FAPEMIG (APQ-02732-15); Conselho Nacional de Desenvolvimento Científico e Tecnológico – CNPq; Coordenação de Aperfeiçoamento de Pessoal de Nível Superior – CAPES/PROEX, and the Biotechnology and Biological Sciences Research Council (BB/K021109/1, BB/G018553, and BB/M023052/1). Funding for JB was also provided by CONFAP - the UK Academies Fellowship (FAPEMIG – APQ-00689-16).
